# Transcriptomic analysis confirms differences among nuclear genomes of cryptic earthworm lineages living in sympatry

**DOI:** 10.1186/s12862-019-1370-y

**Published:** 2019-02-26

**Authors:** Sergei V. Shekhovtsov, Nikita I. Ershov, Gennady V. Vasiliev, Sergey E. Peltek

**Affiliations:** 1 Institute of Cytology and Genetics SB RAS, Pr. Lavrientieva 10, Novosibirsk, 630090 Russia; 2 Institute of Biological Problems of the North FEB RAS, Portovaya St. 18, Magadan, 685000 Russia

**Keywords:** Earthworms, Cryptic diversity, Genetic lineages, Transcriptomics, *Eisenia nordenskioldi*, Species complex

## Abstract

**Background:**

Many earthworm species demonstrate significant cryptic diversity, with several highly diverged mitochondrial lineages found within most of the taxa studied to date. The status of differences between these lineages on the nuclear level is still unclear. Because of widespread polyploidy in earthworms, most studies were limited to two nuclear loci, the ribosomal and the histone clusters. Here we attempted to elucidate the status of a set of genetic lineages within *Eisenia nordenskioldi nordenskioldi*, an earthworm species from Northern Asia with high intraspecific diversity. We performed RNA-seq on an IonTorrent platform for five specimens of this species belonging to five genetic lineages, as well as two outgroups from the family Lumbricidae, the congenetic *E. andrei*, and *Lumbricus rubellus*.

**Results:**

We de novo assembled transcriptomes and constructed datasets of genes present in all seven specimens using broad (ProteinOrtho; 809 genes) and narrow (HaMStR; 203 genes) ortholog assignment. The majority of orthologs had identical amino acid sequences in all studied specimens, which we believe was due to strong bias towards the most conserved genes. However, for the rest of genes the differences among the lineages were lower than those between them and the congeneric *E. andrei*. Both datasets yielded phylogenetic trees with the same topology. *E. n. nordenskioldi* was found to be monophyletic. The differences on the genetic level had no concordance with geography, implying complex history of dispersal.

**Conclusions:**

We found that genetic lineages of *E. n. nordenskioldi* are genetically distinct on nuclear level and probably diverged long ago. Current data implies that they might even represent distinct species within the *E. nordenskioldi* species complex.

**Electronic supplementary material:**

The online version of this article (10.1186/s12862-019-1370-y) contains supplementary material, which is available to authorized users.

## Background

One of the notable paradoxes in earthworm systematics is that well-known and ubiquitous species that were extensively studied for over a century were found to harbor very high cryptic diversity [1]. In the last decade molecular studies revealed that many earthworm species contain two to five cryptic mitochondrial lineages, with about 10–20% of nucleotide substitutions among them (e.g., [2–5]). Different lineages are often found in individuals living in sympatry and forming a morphologically uniform sample.

It is not yet clear what these genetic lineages are. There are two possible extreme possibilities; one is that they represent distinct species that could not be found by morphological analysis due to the paucity of morphological characters in earthworms. The other is that this diversity is the result of yet unknown mitochondrial phenomena that are however accompanied by no significant differences in nuclear genomes. Some molecular genetic studies favor the former hypothesis [6, 7], and others the latter [8, 9]. Moreover, sometimes differences on the nuclear level are found between some lineages within a species but not the others [10–12]. In some cases genetic isolation exists but is not complete [10].

However, the majority of studies cannot be considered conclusive, as they were based on small DNA fragments; current nuclear phylogenetic markers are mostly limited to the ribosomal RNA cluster and short fragments of the histone H3 (~ 350 bp) and H4 (~ 180 bp) genes, which evolve slowly and thus cannot provide reliable phylogenetic resolution at this level. Extensive studies of nuclear divergence are impeded by the lack of suitable primers, and also by the fact that many earthworm species are polyploids [13, 14]. To illustrate, one of the best-studied examples is *Lumbricus rubellus*, a common cosmopolitan species throughout the Northern Paleartic. This species is known consist of at least eight genetic lineages [12]. Studies using microsatellites [15] and histone H3 sequences [12] detected differences in nuclear genomes among the studied lineages of *L. rubellus*. The latter study [12] postulated the existence of seven well-supported cryptic species within *L. rubellus*, however, it should be noted that some of them were distinguished based on as few as two nucleotide substitutions in the H3 gene. In contrast, Giska et al. [8] used the RADseq method on a set of sympatric genetic lineages of *L. rubellus* and found no genetic subdivision on the nuclear level. It is impossible to compare the data obtained in these three studies, because they were made using different methods and on distinct set of lineages.

Transcriptomics, i.e. sequencing of the total mRNA content, can provide us with information on thousands of expressed genes. In contrast to total nuclear genomes that mostly consist of non-coding DNA and mobile elements, much of mRNA data may be phylogenetically informative, and the high number of genes could allow us to test if trees built on different gene datasets are in accordance with each other. There is currently a number of studies on earthworm transcriptomes; while most of them focus on single species, Novo et al. [16, 17] employed transcriptome data to study phylogeny of Hormogastridae, and Anderson et al. [18] investigated relationships among earthworm families. This method has an advantage that datasets from different transcriptomic studies can be combined together.

One of the well-studied cases of cryptic diversity is *Eisenia nordenskioldi*, a Siberian earthworm with a wide distribution ranging from tundra to southern steppes [19]. This species contains two subspecies (the pigmented *E. n. nordenskioldi* and the unpigmented *E. n. pallida*), several races of different ploidy, and at least 15 genetic lineages [20–26]. While the differences among COI gene sequences of these genetic lineages are significant, those within the nuclear ribosomal cluster are less pronounced, and clear phylogeny cannot be resolved using any of these markers [21, 23].

In this study we used the IonTorrent platform to sequence transcriptomes of five *E. n. nordenskioldi* lineages: lineage 3 (south of West Siberia), 7 (Southern Urals), 9 (Northeast Siberia), as well as two yet unnamed lineages from the Altai Mountains. As outgroups, we used two species from the family Lumbricidae, *Lumbricus rubellus* and the congeneric *E. andrei*.

## Methods

### RNA and DNA extraction

**Fig. 1:**
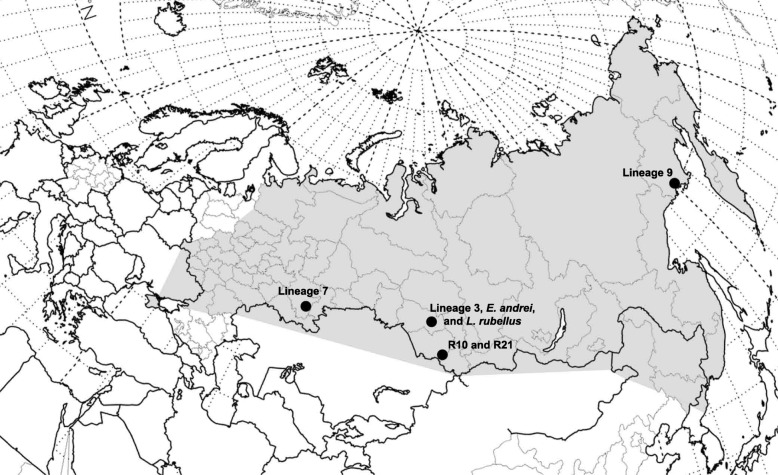
Sampling locations. Shading indicates an approximate range of *E. n. nordenskioldi*

Earthworm individuals were collected in 2016 (Table 1, Fig. 1). Prior to nucleic acid extraction they were kept on filter paper for 3 days. For RNA extraction, worms were washed with distilled water. A fragment of the posterior body part (about 100 μg) was fixed in ethanol for DNA extraction. The middle part of the body including the clitellum (about 100 μg) was excised with scissors and briefly homogenized with a plastic pestle before the addition of 600 μl TRIzol (Sigma-Aldrich, USA). The tube was stirred and 150 μl of chloroform was added. The tube was incubated for 5 min and centrifuged for 10 min at 16000 g at 4 °C in an Eppendorf centrifuge. The supernatant was transferred to a new tube with 150 μl chloroform, stirred, and centrifuged again as described above. This procedure was repeated, then we added 1/2 V of isopropanol to the resulting supernatant, incubated the tube at − 20 °C for 30 min and centrifuged it for 10 min at 12000 g at 4 °C. The pellet was washed twice with 75% ethanol, air-dried and dissolved in DEPC-treated water.

**Table 1:** Summary statistics for transcriptome sequencing and assembly

Sample	Reads	Bases	Ntr	N50	nr	POrtho	HaMStr
E.n.n. lineage 3	1,002,477	267,977,445	41,724	830	17,469	10,574	942
E.n.n. lineage 7	901,691	233,570,944	26,230	787	12,617	8567	851
E.n.n. lineage 9	907,172	217,418,513	35,751	693	13,952	9041	876
E.n.n. R10	762,294	183,220,380	29,961	684	11,937	8056	814
E.n.n. R21	820,821	198,132,912	40,334	733	17,222	10,519	1020
*E. andrei*	1,094,366	273,164,798	41,886	811	19,351	10,673	974
*L. rubellus*	737,035	195,074,949	36,539	829	18,498	9405	1018

E. n. n., *Eisenia n. nordenskioldi*; Ntr, number of assembled transcripts; nr, number of non-redundant proteins; POrtho, number of the detected ProteinOrtho OGs out of a total possible of 20,603; HaMStR, number of the detected HaMStR OGs out of a total possible of 1253

DNA was extracted from the ethanol-fixed samples using silica columns (BioSilica, Novosibirsk) according to the manufacturer’s instructions. The standard COI fragment was amplified using universal primers, the forward LCO1490m (5’-TACTC-AACAA-ATCAC-AAAGA-TATTG-G-3′; modified from [27]), and the reverse primers HCO2198 (5’-TAAAC-TTCAG-GGTGA-CCAAA-AAATC-A-3′; [27]) or COI-E (5’-TATAC-TTCTG-GGTGT-CCGAA-GAATC-A-3′; [28]). After Sanger sequencing, the GenBank database and our own barcoding collection were used for sequence identification. The specimens belonging to the desired COI lineage were selected for transcriptome analysis.

### Preparation and sequencing of libraries

Total RNA was purified using a PureLink RNA Mini Kit (Invitrogen) with on-column DNAse I treatment. RNA quality was checked by Bioanalyzer 2100 with RNA Nano chips. Poly-A RNA was extracted from 10 μg of total RNA using a Dynabeads mRNA Purification Kit (Invitrogen). RNA-Seq library preparation was carried out using an Ion Total RNA-Seq Kit v2 (Life Technologies) according to the manufacturer’s instructions with modifications: chemical RNA fragmentation for 5 min and PNK end repair was used instead of enzymatic fragmentation to increase reproducibility and proportion of long fragments. A total of 50 ng of fragmented mRNA was taken as the template. After 9 cycles of amplification, libraries were purified using 1.5 V of Agencourt® AMPure XP beads. Library fraction of 290–380 bp (median insert size ~ 260 bp) was extracted using LabChip® XT (Perkin-Elmer, USA) DNA 500 chips. The purity and concentration of final libraries were estimated by Bioanalyzer 2100 with DNA High Sensitivity Chip. Templates for Ion Torrent sequencing were prepared by using an Ion PGM™ Template OT2 400 Kit, One-Touch 2, and One-Touch ES systems (Life Technologies). Libraries were sequenced on an Ion PGM machine using Ion318v2 chips deposited at full density following the protocol for 400 bp sequencing supplied by the manufacturer.

### Transcriptome assembly and analysis

Each set of single-end sequence reads was de novo assembled using Trinity (v2.4.0) [29] with default parameters. TransDecoder (v5.0.0; http://transdecoder.github.io) was utilized to predict protein-coding regions in assembled transcripts, also using the matches produced by HMMER hmmscan (v.3.1b2; http://hmmer.org/) against the Pfam (https://pfam.xfam.org/) database and blastp (v 2.7.1+) against the UniProt Swiss-Prot (https://www.uniprot.org/) database as the selection criteria. The redundancy in the set of predicted proteins was reduced using CD-HIT (v4.6) [30] at 0.99 similarity threshold.

Initial orthologous groups (OGs) were determined using two independent algorithms, HaMStR (v13.2.6) [31] as a narrow approach, and a more productive method implemented in ProteinOrtho (v5.16b) [32]. HaMStR was set to search against the Lophotrochozoa HMMs in strict mode using *Helobdella robusta* and *Capitella sp.* as the reference species. ProteinOrtho was run with 1e^− 30^ e-value threshold for matches. Only the OGs represented by all specimens under study were retained; the sequences were aligned with MAFFT (v7.394) [33]. Alignments were strictly trimmed with TrimAl (v1.4.rev22) [34] by sequentially using the ‘strictplus’ and ‘nogaps’ options.

The resulting aligned OGs shorter than 30 bp or showing dissimilarity between any pair of sequences outside the upper 95th percentile were discarded using a bash script. The rest were filtered using PhyloTreePruner (v1.0) [35] on the corresponding ML trees generated with FastTreeMP (v2.1.7) [36]. Phylogenetic trees were reconstructed by MrBayes (v3.2.6) [37] and RaxML (v8.2.12) [38] for the final concatenated datasets. RaxML was ran with parameters ‘-m PROTGAMMAAUTO -f a -N autoMRE’. For MrBayes, we used the following settings: ‘nst=mixed rates=gamma aamodelpr=mixed ngen=1000000’ and controlled the convergence of the runs (standard deviation of split frequencies (ASDSF) < 0.01, potential scale reduction factor (PSRF) close to 1.00, and effective sample size (ESS) > 300).

Functional annotation of the predicted orthologous groups was performed by mapping the abovementioned Pfam and Swiss-Prot hits to the Gene Ontology (GO) categories using built-in Swiss-Prot information and pfam2go data (http://www.geneontology.org/external2go/pfam2go).

For the ProteinOrtho dataset, we also extracted nucleotide data and performed alignment and construction of phylogenetic trees as described above.

## Results

### Transcriptome assemblies and detection of orthologs

In this study we utilized Ion Torrent low-depth transcriptome sequencing to recover the fraction of the most abundant polyadenylated transcripts for further phylogenetic analysis. About 0.75–1 M reads were obtained for each library. The obtained data is summarized in Table 1. De novo assembly by Trinity produced 26,230–41,886 transcripts with the average N50 of 767 bp. A total of 11,937–18,498 non-redundant proteins were predicted for each specimen.

In spite of relatively low sequencing depth, the data was sufficient to extend up to 65–82% of HaMStR orthogroups per library (Table 1). Alternatively, an approximately ten-times broader set of OGs was generated using the more broad ProteinOrtho algorithm. However, only 1085 out of ~ 10,000 ProteinOrtho orthogroups were present in all sequenced samples. At the same time, 544 HaMStR orthogroups overlapped between all samples, comprising at least half of the individually detected OGs.

**Fig. 2:**
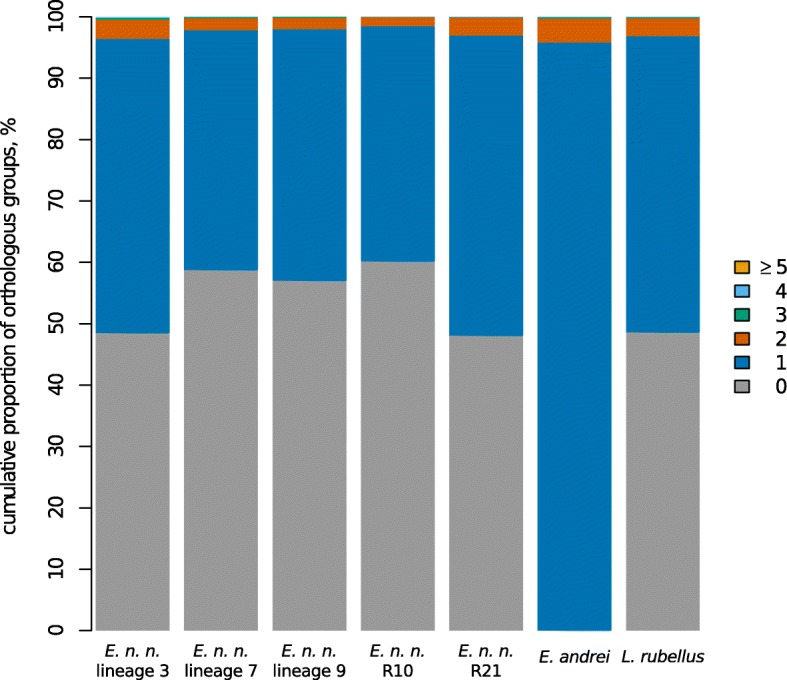
Distribution of orthologous groups in analyzed species by the number of contained paralogous members in relation to *E. andrei*

Since *E. nordenskioldi* includes forms of different ploidy levels, we also estimated the impact of polyploidy on the structure of inferred orthogroups in the analyzed lineages. We performed a similar ProteinOrtho analysis on protein-coding nucleotide sequences to account for synonymous nucleotide substitutions and thereby improve the discrimination of paralogous genes duplicated via polyploidization. However the distribution of orthogroups by the number of included paralogous sequences was essentially the same for each *E. nordenskioldi* lineage and for known diploid species, *E. andrei* (Fig. 2). The vast majority of groups consisted of one-to-one orthologs allowing us to conclude that the impact of possible ploidy on orthology prediction is minor if any.

### Phylogeny of the *E. n. nordenskioldi* complex

**Fig. 3:**
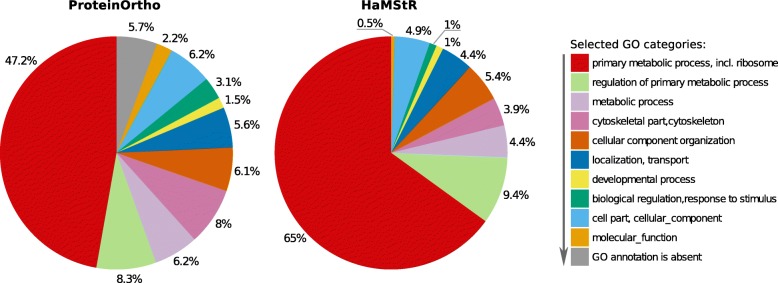
Homology-based functional classification of orthologous groups detected by ProteinOrtho and HaMStR. The arrow in the legend indicates the priority of assigning a category to a gene

To infer phylogenetic relationships among genetic lineages of *E. n. nordenskioldi*, the orthogroups detected by ProteinOrtho and HaMStR were further aligned and extensively filtered, including the removal of OGs with outlying sequence differences (i.e., 5% most differing genes in any pair of samples). This reduced the HaMStR and ProteinOrtho sets down to 203 and 809 OGs, respectively (28,165 and 130,935 amino acids). Most (173) of the remained HaMStR orthogroups were also represented in the ProteinOrtho dataset. According to the homology-based classification of the selected orthologous genes in terms of Gene Ontology, most of them are involved in core metabolic processes or encode ubiquitous structural proteins (Fig. 3), although the functional diversity appeared to be slightly higher in the ProteinOrtho dataset.

**Fig. 4:**
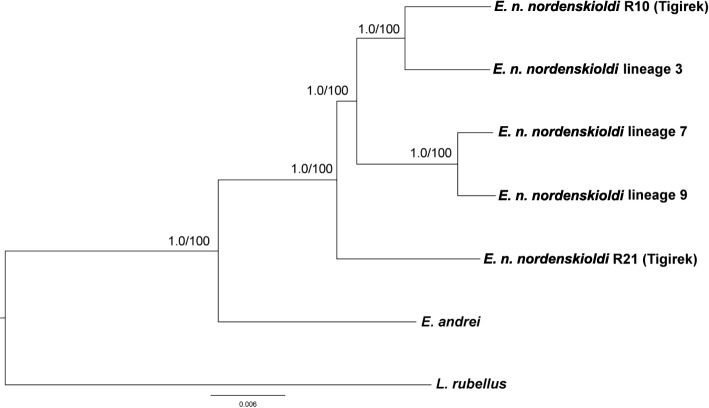
Phylogenetic tree obtained for the HaMStR dataset using the ML algorithm. Numbers near the nodes indicate ML bootstrap value/Bayesian posterior probabilities

To monitor the validity of orthology prediction and phylogenetic inference procedures, we used both ProteinOrtho and HaMStR datasets in combination with Maximum Likelihood and Bayesian methods, implemented in the RAxML and MrBayes programs, respectively. All four variants yielded identical trees (Fig. 4), and the same tree was found for the ProteinOrtho nucleotide data (a total of 390,590 bp). *E. n. nordenskioldi* was found to be monophyletic. One specimen from Tigirek (R21) was the sister group to the rest of *E. n. nordenskioldi* lineages; the rest formed two closely related pairs, i.e. *E. n. nordenskioldi* lineages 7 and 9, and lineage 3 with the other specimen from Tigirek (R10).

### Pairwise distances among the specimens for on the species, genus, and family levels

For the concatenated HaMStR dataset, the average pairwise p-distance among the specimens of the *E. n. nordenskioldi* lineages was 1.73%; among *E. n. nordenskioldi* lineages and *E. andrei*, 3.06%; *E. n. nordenskioldi* lineages and *L. rubellus*, 4.50%. Respective distances for the ProteinOrtho set were somewhat higher: 2.60, 4.75, and 6.87%. The expanded table with distances among the specimens representing individual lineages is given in Table 2. As expected, nucleotide distances were higher; for the ProteinOrtho dataset: 4.02% among specimens of the different *E. n. nordenskioldi* lineages, 7.63% among *E. n. nordenskioldi* lineages and *E. andrei*, 9.96% *E. n. nordenskioldi* lineages and *L. rubellus.*

**Table 2:** Pairwise p-distances among concatenated datasets of the studied specimens

	E.n.n.3	E.n.n.7	E.n.n.9	E.n.n. R10	E.n.n. R21	*E. andrei*	*L. rubellus*
E.n.n.3	–	2.81%	2.80%	1.93%	3.01%	4.73%	6.83%
E.n.n.7	1.87%	–	0.85%	2.72%	3.08%	4.76%	6.87%
E.n.n.9	1.94%	0.55%	–	2.68%	3.08%	4.78%	6.91%
E.n.n. R10	1.27%	1.82%	1.82%	–	3.00%	4.65%	6.81%
E.n.n. R21	1.93%	2.01%	2.03%	2.01%	–	4.83%	6.94%
*E. andrei*	3.04%	2.99%	3.07%	3.04%	3.17%	–	6.84%
*L. rubellus*	4.48%	4.49%	4.50%	4.51%	4.50%	4.35%	–

above diagonal, ProteinOrtho dataset; below diagonal, HaMStR dataset. E.n.n., *Eisenia n. nordenskioldi*

**Fig. 5:**
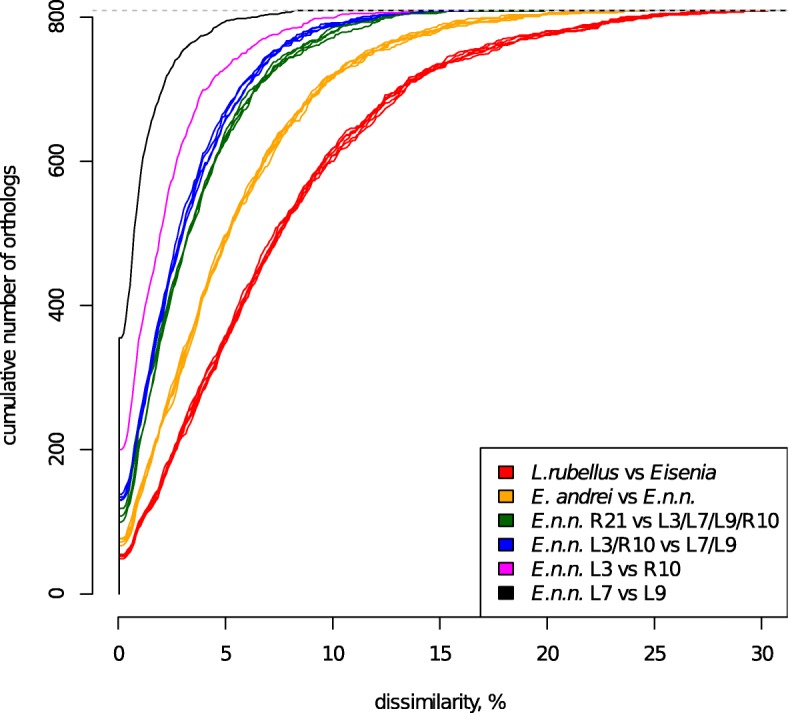
Cumulative distribution of sequence divergence of protein alignments between each pair of samples

However, since average values might not reflect the presence of outlier genes, we constructed a histogram of pairwise distances (Fig. 5 for aa data; Additional file 1 for nucleotide data) for all specimen pairs. As seen on Fig. 5, distributions of pairwise distances between *L. rubellus* and *Eisenia* specimens were almost identical. The same was true for pairwise distances between *E. andrei* and *E. n. nordenskioldi* specimens. Among the latter, there were two outliers, i.e., lineages 7 and 9, which were the two most geographically distant (5300 km) but the most closely related (0.55%/0.85% of differing amino acid sites for HaMStR and ProteinOrtho, respectively). Another pair were the lineage 3 and a specimen from the Altai mountains, with 1.27%/1.93% average distances. All other lineage pairs had very similar distance distributions with pairwise distances of 1.8–2%/2.7–3% (Fig. 5, Table 2).

## Discussion

### IonTorrent transcriptomes

Low-depth transcriptome sequencing is an attractive but challenging way of gathering a representative set of nuclear genes for phylogenetic inference. Insufficient coverage of low expressed genes may hamper the accuracy and diversity of the resulting transcriptome assembly and therefore requires more stringent monitoring and filtering of ortholog assignment. We detected a relatively low overlap of the assembled sequences among the samples, which is most likely explained by the sensitivity of low-depth sequencing to initial differences in transcript abundances among RNA samples arising from species-, stage- and tissue- specificity, as well as differences in sample storage and preparation. However, the chosen approach allowed us to obtain a considerable set of highly expressed transcripts, which was more sufficient for further phylogenetic analysis.

### Differences among expressed genes

In this study we aimed to assess differences among protein sequences on the level of species, genus, and family in earthworms. However, the majority of OGs were identical on the protein level or had sequence divergence below 1% for all studied specimens (Fig. 3). This could be due to several reasons. First, RNA-seq has strong bias towards highly expressed housekeeping genes that are more conserved. Second, our processing retains the genes that are found in the databases of orthologous genes, again keeping the most conserved ones. Third, we performed strict filtering of the alignments thus excluding strongly diverged genes. For these reasons, average pairwise distances among the studies specimens calculated for our datasets are much lower than those that would be observed by comparing the whole genomic data.

These sequences with slow molecular evolution provide almost no information for the phylogenetic questions under study. However, for the rest of the OGs there is a clear trend of increasing sequence distance for the studied specimens from the species to the family level (Fig. 5 and Additional file 1: Figure S1). This sounds trivial, but we should note that phylogenetic trees built using mitochondrial sequences for the family Lumbricidae suggest that the differences among congeneric species are often as high as those among genera, and the expected monophyly for many genera is often lacking [39–41]. However, to our opinion, at least in some cases this is caused by saturation with substitutions resulting from the high rate of mtDNA evolution, not the actual phylogeny of the studied taxa, making large datasets of nuclear genes really promising for elucidating these questions.

### Phylogeny and cryptic lineages of *E. n. nordenskioldi*

The sampled set of *E. n. nordenskioldi* lineages turned out to be monophyletic with respect to *E. andrei*. However, there are certain reservations here. First, we should note that although *E. andrei* and *E. nordenskioldi* belong to the same genus, they are distant relatives. *E. nordenskioldi* is a species of northern Asia, while *E. andrei* is found throughout the world. It would be more sensible to use a more closely related species of the genus *Eisenia* as an outgroup, e.g., an Altai or Ural endemic [19]. Second, we sampled only a few lineages of *E. nordenskioldi*; the complete set could turn out to be much more diverse.

It is noteworthy that the relationships among the lineages were not concordant with geography: the most geographically distant specimens in this study, lineages 7 and 9 were the most genetically closely related (Figs. 4 and 5). Another branch was formed by lineage 3 and the specimen R10 from Tigirek. R21 from Tigirek was the sister group to the rest of *E. n. nordenskioldi* lineages.

The lack of concordance between genetics and geography corroborates the viewpoint that *E. n. nordenskioldi* lineages diverged long ago, before the Pleistocene at least [21], and had a complex history of dispersal, range expansions and contractions. The fact that a specimen from Tigirek was the sister group to the rest of *E. n. nordenskioldi* lineages confirmed our earlier findings that *E. nordenskioldi* from the Altai Mountains has very high morphological and genetic diversity even compared to the already remarkable diversity of this species on the plains of Northern Eurasia [22, 23, 25]. We might thus hypothesize that the Altai Mountains are the center of origin of *E. n. nordenskioldi*; however, we would need further studies to clarify this point.

### Polyploidy in *E. n. nordenskioldi*

It is well known that many earthworm species are polyploids or even consist of a set of forms with different ploidy [42, 43]. The latter is true for *E. nordenskioldi*, which is known to have di-, tetra-, hexa-, hepta-, octa-, and probably even decaploid forms [13, 14, 44]. Nothing is known on the relationships among chromosome races and DNA lineages in *E. nordenskioldi*; it is also unclear if these are allo- (i.e., among genetically divergent populations/lineages) or autopolyploids. Given the prevalence of polyploids in this species, we could have assumed that some of the lineages sampled in this study were polyploids. *E. andrei*, however, is known to be a diploid [42, 43]. Thus, we used this species as a reference: if any other specimen in our sample would demonstrate an increased proportion of two or more orthologs to single *E. andrei* genes, we could state that it is a polyploid.

We found that majority of *E. andrei* transcripts had either one or zero orthologs in transcriptomes of different *E. n. nordenskioldi* lineages, as well as *L. rubellus* (Fig. 2). Only a minor part of orthogroups contained two or more members from the same species, and their proportion was uniform in all specimens. Thus we should conclude that we failed to detect any clear signs of polyploidy within the studied sample.

We should note that this is not a final verdict on this issue. If this was the case of recent autopolyploidy, no substitutions among the paralogs would have accumulated. In allopolyploids, homeologs often have different levels of expression, and many of them become silenced [45], so we would often detect transcripts from only one paralog. We find the latter suggestion particularly probable.

There could also be some computational challenges. Even after the removal of redundancy in transcriptome assemblies, many genes are still represented by several isoforms or vice versa, by transcripts fragmented into several partial sequences. Moreover, nearly identical gene duplications are likely to be assembled into a single sequence. Thus, the proportion of detected paralogous sequences itself cannot be considered a reliable indicator of ploidy level.

## Conclusions

Our results suggest that genetic lineages of *E. n. nordenskioldi* are indeed distinct genetic entities. Although we cannot exclude the possibility of hybridization (other molecular genetic methods are required for that), the studied lineages are sufficiently diverged genetically even when they are found in sympatry. However, the degree of sequence divergence is lower compared to that between *E. n. nordenskioldi* lineages and *E. andrei*. It might be possible that these genetic lineages can be treated as distinct species within the *E. nordenskioldi* species complex. However, more dense sampling with more lineages, several specimens of a single lineage to study variation within lineages, and closer outgroups are required to make such conclusions.

## Additional file

Additional file 1: Cumulative distribution of sequence divergence of nucleotide alignments between each pair of samples. (PDF 17 kb)
