# Supplementary figures and images for: Transcriptomic analysis confirms differences among nuclear genomes of cryptic earthworm lineages living in sympatry
Source: BMC Evol Biol. 2019 Feb 26;19(Suppl 1):50. doi: 10.1186/s12862-019-1370-y (PMC6391759; doi:10.1186/s12862-019-1370-y)

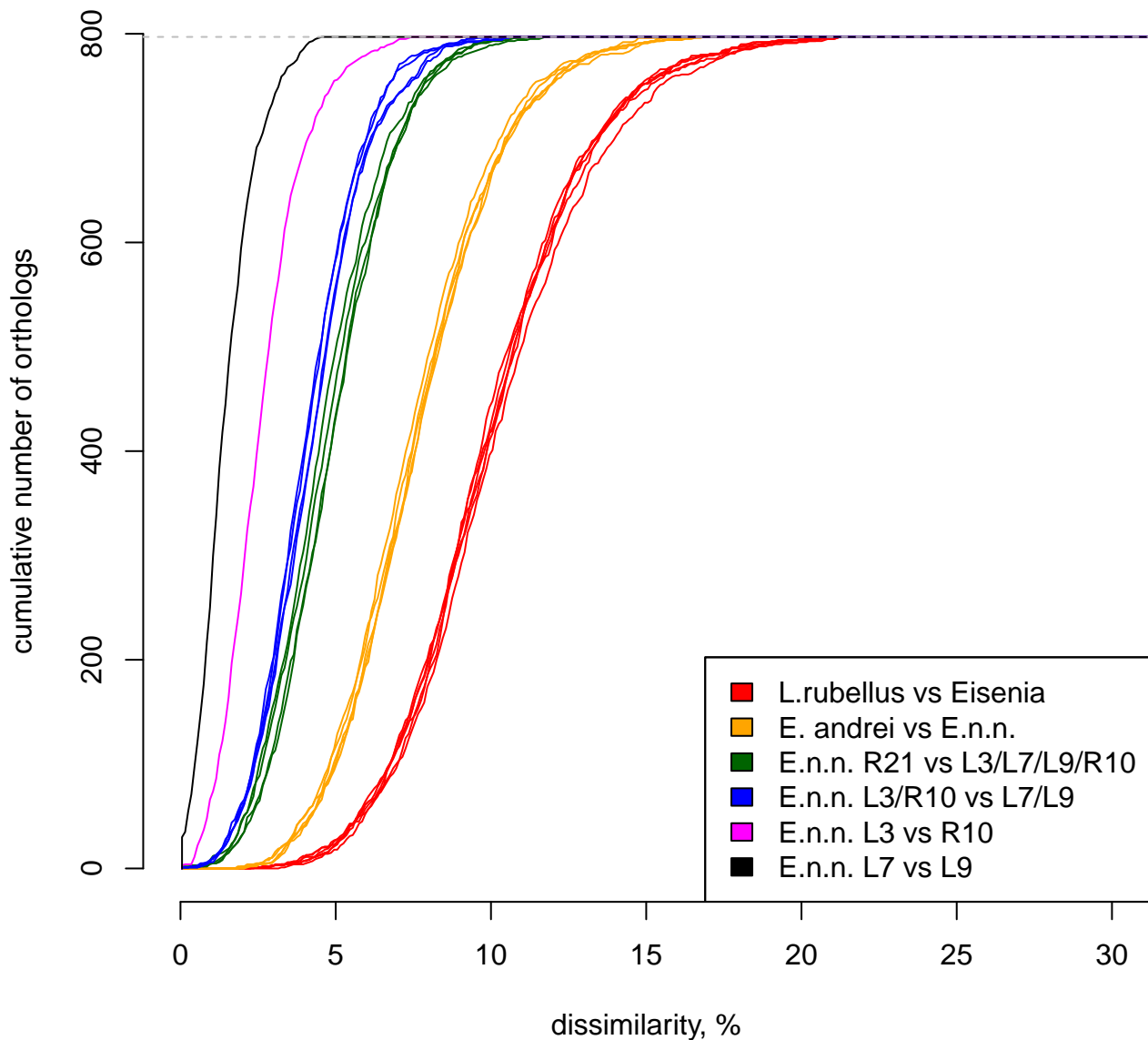

Supplement: Supplementary file 1 — Cumulative distribution of sequence divergence of nucleotide alignments between each pair of samples. (PDF 17 kb) [file 12862_2019_1370_MOESM1_ESM.pdf]
